# Conspicuous Smooth and White Egg-Shaped Sulfur Structures on a Deep-Sea Hydrothermal Vent Formed by Sulfide-Oxidizing Bacteria

**DOI:** 10.1128/Spectrum.00955-21

**Published:** 2021-09-01

**Authors:** Marit R. van Erk, Viola Krukenberg, Pia Bomholt Jensen, Sten Littmann, Dirk de Beer

**Affiliations:** a Max Planck Institute for Marine Microbiology, Bremen, Germany; b Montana State University, Bozeman, Montana, USA; c Aarhus University, Aarhus, Denmark; University of Minnesota

**Keywords:** sulfide oxidation, *Arcobacter*, sulfur filaments, hydrothermal vent

## Abstract

Conspicuous egg-shaped, white, and smooth structures were observed at a hydrothermal vent site in the Guaymas Basin, Gulf of California. The gelatinous structures decomposed within hours after sampling. Scanning electron microscopy (SEM) and light microscopy showed that the structure consisted of filaments of less than 0.1 μm thickness, similar to those observed for “*Candidatus* Arcobacter sulfidicus.” SEM-energy-dispersive X-ray spectroscopy (EDS) showed that the filaments were sulfur rich. According to 16S rRNA gene amplicon and fluorescence *in situ* hybridization (FISH) analyses, *Arcobacter*, a sulfide oxidizer that is known to produce filamentous elemental sulfur, was among the dominant species in the structure and was likely responsible for its formation. *Arcobacter* normally produces woolly snowflake like structures in opposed gradients of sulfide and oxygen. In the laboratory, we observed sulfide consumption in the anoxic zone of the structure, suggesting an anaerobic conversion. The sulfide oxidation and decomposition of the structure in the laboratory may be explained by dissolution of the sulfur filaments by reaction with sulfide under formation of polysulfides.

**IMPORTANCE** At the deep-sea Guaymas Basin hydrothermal vent system, sulfide-rich hydrothermal fluids mix with oxygenated seawater, thereby providing a habitat for microbial sulfur oxidation. Microbial sulfur oxidation in the deep sea involves a variety of organisms and processes and can result in the excretion of elemental sulfur. Here, we report on conspicuous white and smooth gelatinous structures found on hot vents. These strange egg-shaped structures were often observed on previous occasions in the Guaymas Basin, but their composition and formation process were unknown. Our data suggest that the notable and highly ephemeral structure was likely formed by the well-known sulfide-oxidizing *Arcobacter*. While normally *Arcobacter* produces loose flocs or woolly layers, here smooth gel-like structures were found.

## INTRODUCTION

The release of hydrothermal fluids in oxic seawater generates strong thermodynamic disequilibria that fuel the abundant chemoautotrophic microorganisms typical for hydrothermal vent systems. Hydrothermal fluids are generally highly reduced, oxygen-free, and enriched in compounds such as CO_2_, H_2_S, CH_4_, H_2_, and iron (1). Sulfide, as a common constituent of hydrothermal fluids, is a source of energy for chemosynthesis (2). Microbial sulfur oxidation rarely proceeds directly to sulfate but results in a range of oxidized sulfur intermediates, including elemental sulfur and polysulfides (3).

Sulfur oxidizers are well known for their production of elemental sulfur, which is either internally stored or excreted. Produced elemental sulfur has been observed in the form of globules and filaments and can serve as an energy reserve. The excretion process can form thick white mats within a relatively short period of time (4). It has been suggested that the sulfur filaments can be used by organisms as an anchor to position themselves optimally in the chemical gradients present in their habitat (5). An example of a reported responsible organism (both *in situ* and in laboratory incubations) is the epsilonproteobacterium *Arcobacter* (6, 7). Material discovered at several hydrothermal vent sites appeared similar to filamentous sulfur formed by a coastally derived organism, for which filamentous sulfur formation was first described (6, 8), which led to the suggestion that microbial filamentous sulfur is a common product in hydrothermal environments (7). Especially distinct examples are those of the so-called snowblower events. Snowblower events were described as the release of flocculent white bacterial mat fragments into the seawater by venting fluids after eruption (e.g., reference 8).

Essentially all marine sediments harbor sulfur oxidizers, including a large diversity of *Alphaproteobacteria*, *Gammaproteobacteria*, and *Epsilonproteobacteria* (reviewed in reference 9). Well-known forms of sulfur excreted by aerobic marine sulfur oxidizers include white mats (e.g., reference 4). Such mats were described to be gelatinous, mushroom-like (10), consisting of cotton-ball precipitates (11), or rather consisting of the producing organisms within a matrix of sulfur-rich mucous (12).

Here, we report on conspicuous, unusually smooth, white gelatinous egg-shaped structures with a diameter of several centimeters that were observed in the hydrothermal vent system of the Guaymas Basin, Gulf of California. We used microscopy, microsensor measurements, sediment extraction methods, and microbial community composition analyses to describe the sampled structure and the sulfide-oxidizing process as well as determine the inhabiting microorganisms.

## RESULTS

### Observations.

**FIG 1:**
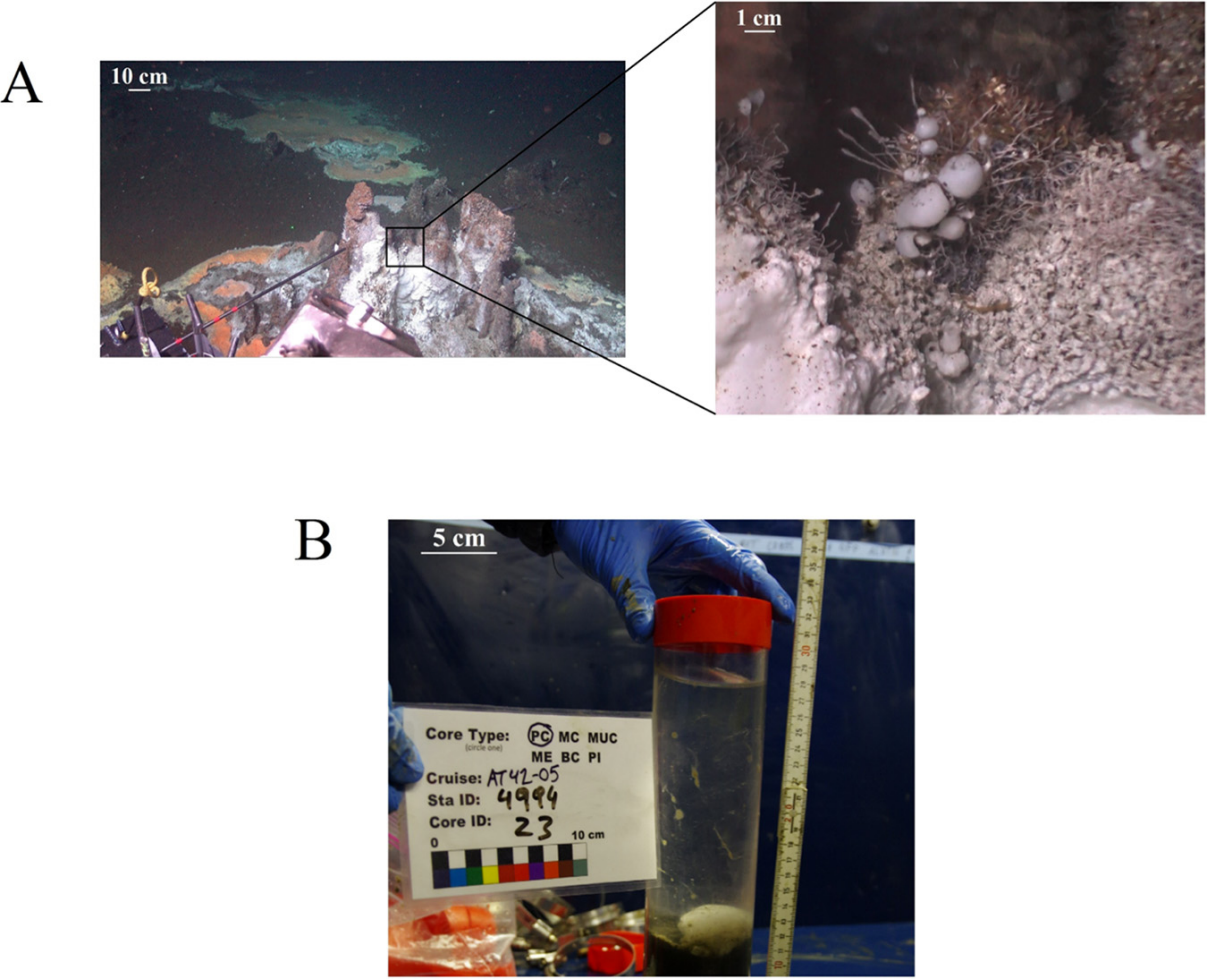
The structures on a hydrothermal vent site at Cathedral Hill (A) and the sampled structure on board of the ship (B). Structures covered the smoker and were abundant on surrounding sediments. The structure shown in panel B was sampled from the surrounding sediments.

White gelatinous flat mats and egg-shaped structures were observed on and next to a hot smoker at Cathedral Hill, Guaymas Basin (Fig. 1A). The semitransparent egg-shaped structures were up to several centimeters in diameter. Some were also growing on tube worms. The unusual smooth gelatinous appearance made the structures very notable.

A structure next to the hot smoker was sampled intact together with the underlying sediment using a push core. The structure had a diameter of a few centimeters (Fig. 1B) and likely recently fell of the hot smoker or tube worm that it was attached to. *In situ* sediment temperatures were 13°C at 5 cm depth and increased up to 99°C at 50 cm depth. Microsensor measurements and subsequent subsampling for the other analyses were conducted before the structure fell apart within a few hours.

### Microsensor measurements.

**FIG 2:**
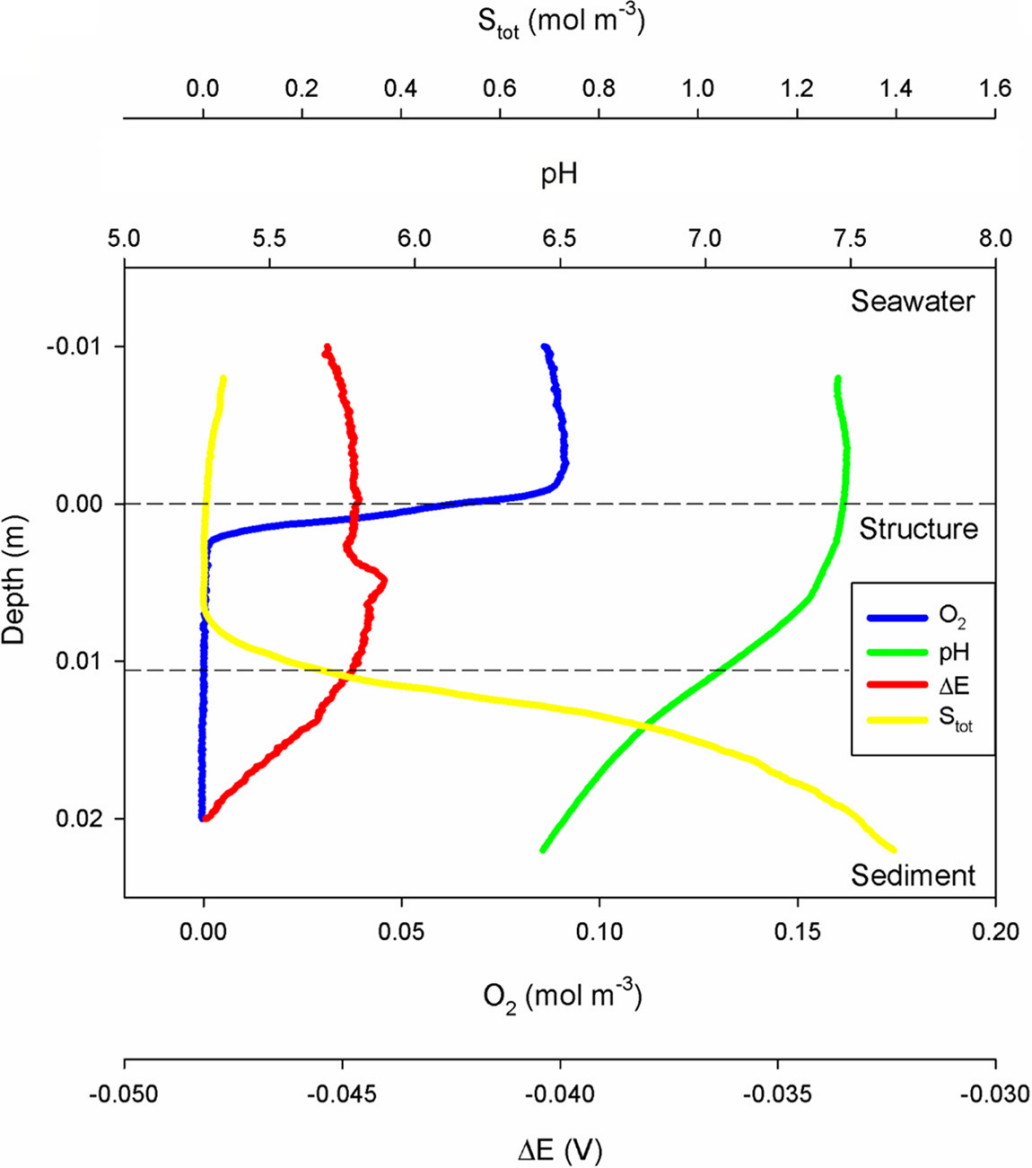
Microsensor profiles measured on the ship from the overlying seawater through the structure (0.00- to 0.01-m depth) into the sediments. Oxygen (in blue) only penetrated a few millimeters into the structure from above, while sulfide (in yellow) penetrated from below. Both were consumed within the structure. No notable change in the electric potential (ΔE; in red) was observed. pH (in green) was stable within the seawater and decreased with depth within the structure and sediment.

Microsensor profiles showed some distinctive features within the structure compared to the overlying seawater and underlying sediment (Fig. 2). Oxygen diffused into the structure from the overlying seawater and penetrated a few millimeters. Sulfide diffused into the structure from the underlying sediment. The oxygen and sulfide profiles showed consumption of both compounds within the structure. However, oxygen and sulfide were consistently spatially separated by 0.5 cm. Hence, sulfide consumption occurred without oxygen being involved.

The pH values in the overlying seawater were stable (7.5), but pH values gradually decreased with depth within the structure and underlying sediment.

The electric potential was negative throughout the measured interval and only showed small differences (<0.005) between the seawater, structure, and sediment.

### Microscopic observations.

**FIG 3:**
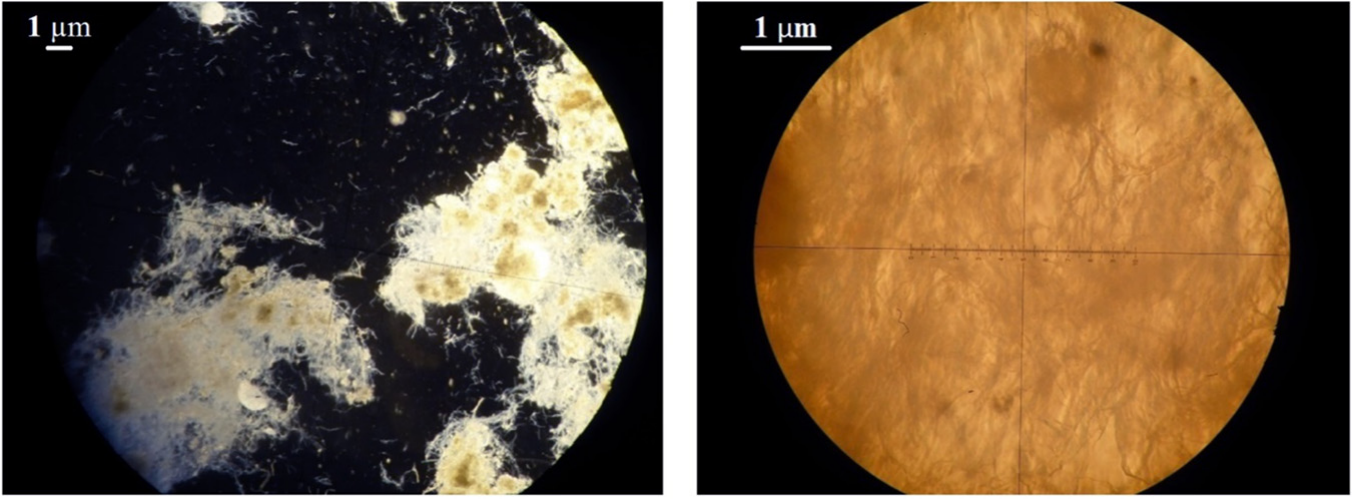
Light microscope images showing a mesh of white filaments with magnifications of ×100 (left) and ×400 (right). The length and thickness of the filaments are variable.

**FIG 4:**
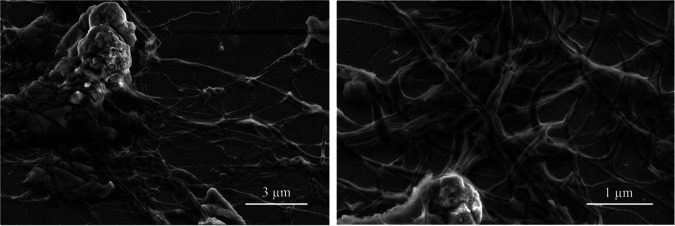
Secondary electron (SE) micrographs from SEM of the filaments. Filaments have a diameter of 0.1 μm or less. No septa are visible.

The structure was composed of a fibrous mesh of irregular whitish filaments (Fig. 3) with variable length and thickness of maximum 0.1 μm (Fig. 4). Upon squeezing between slide and coverslip, the filaments fragmented and completely disappeared. The filaments were rather electron transparent, and no septa were visible (Fig. 4).

### Elemental composition.

The white appearance of the structure indicated sulfur accumulation. The dissolution of the filaments in methanol further suggested the presence of sulfur. Iron concentrations, determined by sediment extraction, were low (<0.1%).

**FIG 5:**
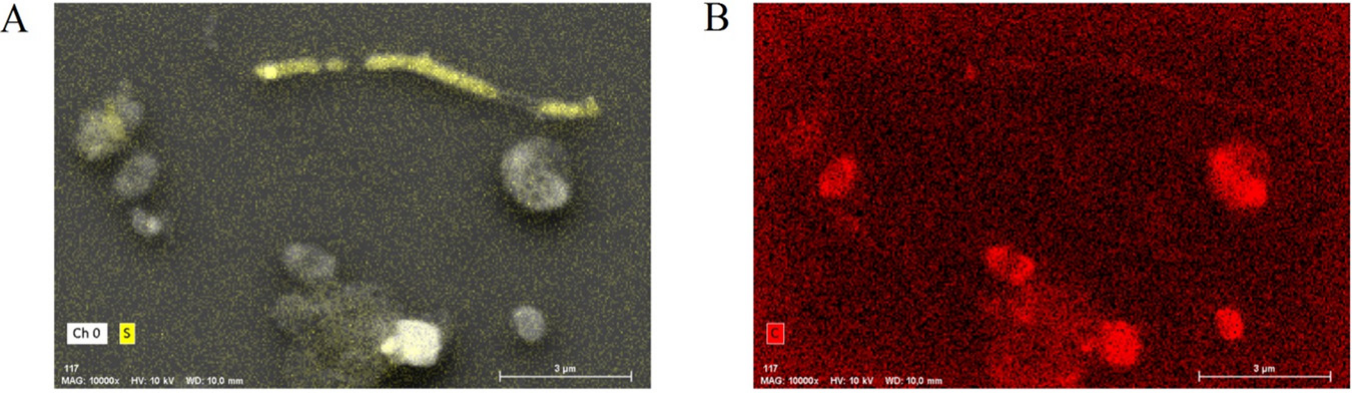
SEM-EDS images showing that filaments (top right) are enriched in sulfur (A) but low in carbon (B) compared to cells. Yellow indicates the presence of sulfur (A), and red indicates the presence of carbon (B). The intensities of these colors in the elemental mapping images are correlated to the abundance of these elements in the sample in mass percent.

The elemental composition, as determined by combined scanning electron microscopy (SEM) and energy-dispersive X-ray spectroscopy (EDS), showed the filaments to be sulfur rich (Fig. 5A), while other elements, such as carbon, were not enriched (Fig. 5B).

### Microbial community composition.

The analysis of the microbial community inferred from 16S rRNA gene amplicon sequencing indicated the prevalence of bacteria (96%) composed of primarily *Epsilonproteobacteria* (67%), with smaller contributions of *Gammaproteobacteria* (8%) and Deltaproteobacteria (6%). Within the *Epsilonproteobacteria*, sulfide-oxidizing genera *Arcobacter* and *Sulfurimonas* each accounted for about half of the reads. *Sulfurovum*, *Desulfobulbaceae*, *Beggiatoaceae*, and *Methanomicrobia* were detected at a low overall frequency (<2%).

On the level of amplicon sequence variants (ASVs), the 15 most frequent ASVs (detected with >300 reads) accounted for about 67% of all reads and were dominated by roughly equal numbers of *Arcobacter* and *Sulfurimonas*. Other taxa (<2%) represented among the most frequent ASVs were *Methylococcales*, *Desulfobulbaceae*, ANME-1 (*Methanomicrobia*), *Sulfurovum*, and *Bacteroidetes.*

Bright-field and epifluorescence microscopy on a nucleic acid-stained sample visualized cells associated with the filamentous sulfur structure.

**FIG 6:**
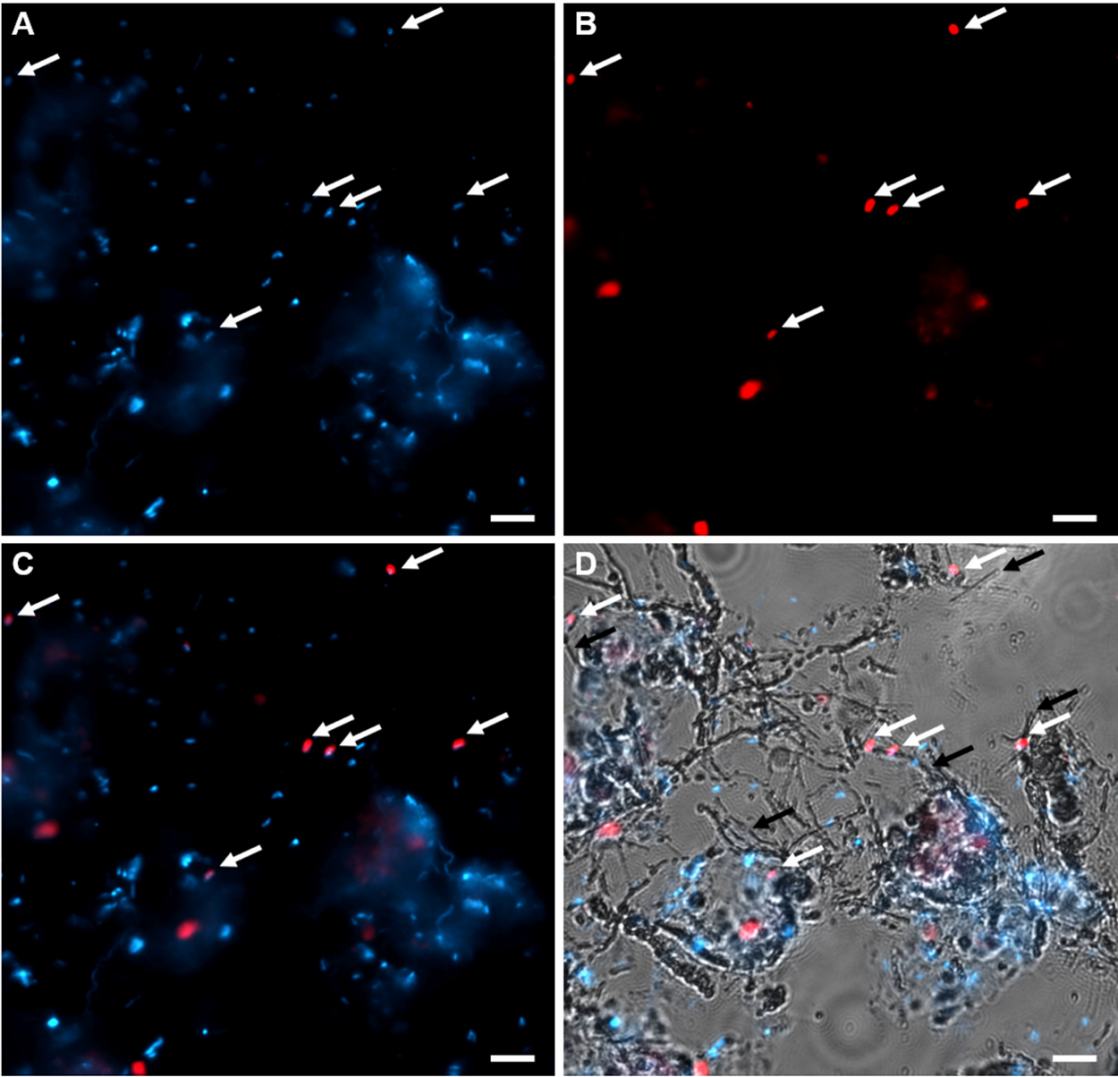
Fluorescence micrographs of cells and filaments. Cells stained with DAPI in blue (A) and cells labeled with CARD-FISH using probe Arc94 targeting *Arcobacter* in red (B). Overlay of DAPI and CARD-FISH signals (C) and fluorescence channels overlaid with bright-field image showing the filaments (D). White arrows indicate cells labeled with probe Arc94 while black arrows highlight filaments in the proximity of labeled cells. Scale bar, 5 μm.

Catalyzed reporter deposition fluorescence *in situ* hybridization (CARD-FISH) with the *Arcobacter*-specific oligonucleotide probe Arc94 (13) confirmed the presence of *Arcobacter* cells, which were observed as coccoid to oval-shaped single cells often connected to the sulfur filaments (Fig. 6).

## DISCUSSION

The conspicuous smooth and gelatinous transparent structures that we report on here have been observed previously (A. Teske and S. Wankel, personal communication) but were not further investigated. Other remarkable sulfur structures have been observed previously in deep sea environments. Mushroom-like mats produced by *Thiobacterium*, a gammaproteobacterium, were reported in sulfidic marine habitats (10). The structures in the mats were much less smooth and smaller than the structures that we report on here and were inhabited by different organisms. Also, white mats consisting of small granules were observed at the surface of a brine seep in the Eastern Mediterranean. With a similar fibrous microstructure and inhabiting organism (likely organisms related to “*Candidatus* Arcobacter sulfidicus”) (11), these structures were irregular, consisting of cotton ball-like precipitates instead of the smooth structure described here. Other sulfur-rich structures include flocculent material composed of filaments observed in a laboratory reactor (6), which was similar to flocculent material released during snowblower events, and hydrothermal filamentous sulfur mats (4), of which a morphologically comparable mat was dominated by *Arcobacter* (12). In the latter, well-described example, the organism was similar on a 16S rRNA gene level to the *Arcobacter* sp. found in this study. However, the structure studied here had a different macroscopic appearance, probably because formation conditions differed.

PCR-based community analyses suffer several well-known biases and should be considered as indicative rather than quantitative (14, 15). Our analysis of the microbial community indicated that 16S rRNA gene amplicons related to *Arcobacter* and *Sulfurimonas* accounted for a substantial proportion. However, as *Arcobacter*, but not *Sulfurimonas*, is known to produce filamentous sulfur, we focused further on *Arcobacter*. The detected *Arcobacter* population was dominated by a single ASV, which was similar to environmental sequences recovered from deep sea and seep environments, and the 16S rRNA gene of “*Candidatus* Arcobacter sulfidicus,” a sulfide oxidizer known to form filaments of elemental sulfur (7). The presence of *Arcobacter* cells was detected with CARD-FISH, which indicated that cells were associated with the sulfur filaments. Although the microstructure of the filaments observed by light microscopy and SEM was very similar to the microstructures that “*Candidatus* Arcobacter sulfidicus” produced in laboratory cultures (7), the macroscopic structure was entirely different. The formation of filaments is thought to help the cells find and maintain an optimal position in opposing chemical gradients of oxygen and sulfide (5). Similar to the well-known “run and tumble” behavior of swimming bacteria to orient themselves optimally in a substrate gradient (16), sudden direction changes of cells growing a sulfur wire explain the strange curvature of the sulfur filaments (Fig. 4).

The SEM-EDS analysis showed that the filaments consist of almost pure sulfur, without an important contribution of metals or carbon. This was confirmed by the observed disappearance of filaments after exposure to methanol, which dissolves elemental sulfur. Our data suggest that the sulfur filaments are likely produced by bacteria related to *Arcobacter*, which live at the interface between oxygen and sulfide and produce elemental sulfur by aerobic sulfide oxidation, as found for “*Candidatus* Arcobacter sulfidicus” (7).

Also, *Sulfurimonas* seems abundant in the structure as observed by microbial community analysis. *Sulfurimonas* can oxidize a range of reduced sulfur compounds, such as sulfide, elemental sulfur, and thiosulfate (17). *Sulfurimonas* is able to oxidize sulfide, usually completely, to sulfate (17) and produce crystalline sulfur only as intermediate product under low-oxygen conditions (18). We therefore regarded it likely that *Arcobacter* is responsible for the formation of the abundant and structured filaments.

However, the sulfide consumption observed within the structure cannot be explained by the oxidative activity of *Arcobacter*, as *Arcobacter* needs an overlap of oxygen and sulfide to perform sulfide oxidation. Other processes were considered to explain the gap between the oxic and sulfidic zone, e.g., oxidation by nitrate, Fe(II), nitrate-storing *Beggiatoa*, or cable bacteria.

The giant vacuolated *Gammaproteobacteria* of the family *Beggiatoaceae* form mats on sulfidic sediments. The family contains a variety of metabolic potential, with autotrophic, mixotrophic, and heterotrophic organisms being isolated (19–21). *Beggiatoa* have vacuoles in which they can store nitrate in millimole per liter concentrations (22), which allows them to oxidize sulfide and survive for days to weeks under anoxic conditions (23). The first step of sulfide oxidation leads to the formation of elemental sulfur (24), which can be stored internally, and is used as an electron donor when the external sulfide concentration decreases.

Another sulfide oxidizer that thrives in sediments with separated oxic and sulfidic zones is a filamentous *Desulfobulbaceae* (also referred to as “cable bacteria”) (25). Electric currents couple the sulfide oxidation to the reduction of oxygen (26) or nitrate (27) through microbial cables stretching between the different zones (25). Cable bacteria can be found in a wide variety of environments (28), but these clearly distinguishable and robust filaments were not observed in the investigated structure. Also, sequences related to *Desulfobulbaceae* were only detected at low frequency.

Such indirect oxidation by oxygen is unlikely, as the stoichiometry of sulfide and oxygen fluxes (S_tot_:O_2_ was approximately 5) does not match with aerobic sulfide oxidation to elemental sulfur [S(0)] with a stoichiometry of 2 (O_2_ + 2 HS^−^ → 2 S(0) + 2 OH^−^). Thus, the sulfide consumption cannot be explained by the oxygen flux. Moreover, aerobic sulfide oxidation of HS^−^ (the dominant sulfide species at pH 7.5) to elemental sulfur would lead to a pH maximum where sulfide disappears, which was not observed.

Also, sulfide oxidation by nitrate is not responsible for the observed sulfide consumption in our structure. The highest possible nitrate flux from the water column to the sulfidic zone is 5.1 × 10^−9 ^mol m^−2^ s^−1^, 2 orders of magnitude too low to explain the observed sulfide consumption. Nitrate storing giant *Beggiatoa* were highly abundant in Guaymas Basin sediments but were not observed inside the structure by microscopy. Furthermore, sequences affiliated with *Beggiatoaceae*, the family containing the genus *Beggiatoa*, were detected with low frequency in the amplicon data set.

Further cable bacteria that directly transfer electrons from sulfide to oxygen via electrical conductance (25, 26) can be excluded as an explanation for the observed gap. Both by light microscopy and SEM, the characteristic filaments with nodes and longitudinal thin ridges typical for cable bacteria (25) were not observed, and observed filaments were much too thin (0.1 μm instead of the typical 0.4 to 1.7 μm for cable bacteria [29]). Furthermore, the cable bacteria are highly robust, while the investigated filaments disappeared when squeezed under microscopic cover glass. The filaments almost completely consisted of sulfur, a poor conductor. Also, the typical pH profile for cable bacteria, with a pH peak in the oxic zone (26), was not observed, and furthermore electric potential measurements differed from those typical for the presence of cable bacteria (30).

The observed sulfide consumption in our laboratory experiments may be explained by the incubation conditions that differed from the *in situ* situation. *In situ*, the structures grow attached to branches of tube worms exposed to oxygen and sulfide from the surrounding water; hence, oxygen and sulfide are provided from the same direction. Indeed, sulfide is abundant in vent fluids of the Guaymas Basin (31). Because *in situ* sulfide and oxygen penetrated the structure from the same direction, sulfide would be rapidly consumed at the surface by sulfide-oxidizing bacteria and be absent inside the structure. Under such conditions, the sulfur filaments would not dissolve. The situation of oxygen and sulfide coming from the same direction may have also led to the smooth appearance instead of woolly flocs. *In situ*, the concentration of oxygen probably exceeded that of sulfide, as upon retrieval, the water phase was no longer sulfidic. Thus, *in situ* sulfide was efficiently oxidized to sulfur at the surface and could not penetrate the structure. In contrast, in our laboratory experiment, the sulfur structure was located in opposing gradients of sulfide from the sediment and oxygen from the water column. Hence, the filaments of sulfur were exposed to sulfide that penetrated from below, which would react with the filaments before meeting the oxygen penetrating the structures from above. Because sulfide penetrated the structure from the sediments below, it could react with the filaments to form polysulfides (n/8 S_8_ + HS^−^ → S_n+1_^2−^ + H^+^ [32]), resulting in dissolution of the filaments and decomposition of the structure within hours. Our H_2_S microsensors do not detect polysulfides. In opposing gradients, as is normally the case in other environments, sulfur filaments are constantly exposed to sulfide, and the cells will have a more complicated pattern to find the optimal location in the oxygen-sulfide gradients, as the exposed surfaces are limited in either oxygen or sulfide. This might lead to a more irregular flocculent and woolly assembly of the individual filaments forming the structure.

## MATERIALS AND METHODS

### Site description and sample collection.

Sampling took place during a research cruise with RV *Atlantis* and DSV *Alvin* in the Guaymas Basin (Gulf of California) in November 2018. Structures were observed on and next to a hot smoker at the Cathedral Hill hydrothermal vent system (27°00.696 N, 111°24.254 W) (33). One of the structures next to the hot smoker was sampled by the *Alvin* submersible using a push corer. On board, the push core with the structure was brought to a cold room (4°C) for description of sediment characteristics.

### Microsensor measurements.

The push core with the structure was placed in a water bath, which was set at a constant temperature of 3°C. Microsensor profiles for O_2_, pH, H_2_S, and electric potential were measured through the structure, overlying seawater, and underlying sediment. The microsensors for O_2_, pH, H_2_S, and electric potential were produced as described previously (30, 34–36). The interface between the structure and the seawater was set as zero position. Depth profiles were measured using a micromanipulator equipped with a motor. The O_2_ microsensors were 2-point calibrated with air-saturated seawater (100%) and 1 mol liter^−1^ Na ascorbate solution, pH 11 (0%). The pH microsensors were calibrated in standard buffers. The H_2_S microsensors were calibrated by incremental addition of a Na_2_S stock solution to acidified seawater (pH < 2). Concentrations of total sulfide (S_tot_) (S_tot_ = H_2_S + HS^−^ + S^2−^) were calculated using the corresponding H_2_S concentrations and pH values (34) for each depth, using a pK1 of 6.635 (37). Subsamples for microscopic, chemical, and community analyses were taken after microsensor measurements were finished.

Fluxes of oxygen and sulfide were calculated by multiplication of the molecular diffusion coefficient of oxygen (D_0_) with the porosity of the structure (38), resulting in fluxes of 4.9 × 10^−8^ mol m^−2^ s^−1^ for oxygen [Ds(O_2_) = 1.98 × 10^−9^ m^2^ s^−1^] and 2.2 × 10^−7^ mol m^−2^ s^−1^ for sulfide [D_s_(S_tot_) = 0.64×D_s_(O_2_)] (39, 40), respectively. The highest possible nitrate flux was calculated using the combined nitrate and nitrite concentration in bottom water of 20 μmol liter^−1^ (33) and a D_0_ of 1.61 × 10^−9^ m^2^ s^−1^ (41).

### Microscopy and elemental analysis.

Subsamples of the structure were taken for light microscopy, scanning electron microscopy (SEM), and energy-dispersive X-ray spectroscopy (EDS) analyses. Light microscopy was conducted on board. Samples for SEM were fixed on board using a fixative solvent of paraformaldehyde 2.5%/glutaraldehyde 2.5%/Na-cacodylate 0.1 mol liter^−1^. The objective of fixation is to retain cellular components in their native compartments and to present cells with a distinct and microscopically detailed appearance. The sample was rinsed/diluted in water 1:1, and one drop of this solution was added to a fresh drop of water on a piece of silicon wafer and left for drying out at room temperature. SEM analysis was performed on a Verso 3D, with the following scanning parameters: 5 kV and 13 pA with an angle of 45 degrees.

For SEM-EDS, the samples were prepared on chips of silicon wafer material. Secondary electron micrographs were recorded using an FEI Quanta 250 FEG (Thermo Fisher Scientific, Eindhoven, The Netherlands) scanning electron microscope with an acceleration of 2 and 5 kV for the electron beam. Energy-dispersive X-ray spectroscopy was performed with an accelerating voltage of 10 kV on the SEM and Bruker EDS double detector system equipped with XFlash 6/30 detectors (Bruker Nano GmbH, Berlin, Germany) with an energy resolution of <123 eV at MnKα. EDS data were processed with the Bruker Quantax Esprit software package.

### Solid-phase iron extractions.

Subsamples for solid-phase iron extractions were fixed in 5% (wt/vol) ZnAc and stored at −20°C on board. The subsamples were transported cooled and were stored at −20°C in the home laboratory. Extraction of solid-phase iron occurred on around 50 mg material for 0.5 h using 0.5 mol liter^−1^ HCl. Solid-phase iron concentrations were determined on the filtered (0.2 μm polytetrafluoroethylene [PTFE] syringe filters) extract using the ferrozine method (42).

### DNA extraction, 16S rRNA gene amplicon sequencing, and bioinformatic analysis.

A subsample was preserved for DNA extraction by spinning down 1 ml material, removing the supernatant, and storing the pellet at −20°C. An aliquot was used for DNA extraction with the FastDNA spin kit for soil (MP Biomedicals) following the manufacturers guidelines, and DNA was quantified with the Qubit assay. The 16S rRNA gene of *Archaea* and *Bacteria* was amplified using the universal primers 515F (5′-GTGYCAGCMGCCGCGGTAA-3′) and 806R (5′-GGACTACNVGGGTWTCTAAT-3′) following the Earth Microbiome protocol (43–45). PCRs consisted of 10 μl Invitrogen Platinum *Taq* II 2X master mix, 0.5 μl 515F primer (10 μmol liter^−1^), 0.5 μl 806R primer (10 μmol liter^−1^), 9 μl nuclease-free water, and 5 μl of template DNA (5 ng μl^−1^). PCR was performed with the following thermocycler conditions: 94°C for 3 min followed by 28 cycles of 94°C for 45 s, 50°C for 60 s, and 72°C for 90 s and a final elongation step at 72°C for 10 min. PCR products were checked for the expected length on an agarose (1%) gel and were purified using AMPure XR beads (Beckman Coulter) following the manufacturers protocol. Subsequently, the gene amplicons were prepared for Illumina MiSeq sequencing following Illumina’s 16S library preparation protocol as previously described (46). Illumina MiSeq sequencing with 2 × 250 bp paired-end read chemistry was performed by Laragen Inc. (Culver City, CA). Demultiplexed sequence reads were processed using QIIME 2 (version 2020.2) (47). In short, primer sequences were removed using cutadapt with an error rate of 0.12, and reads were further processed in DADA2. Forward and reverse reads were truncated to 140 bp, and filtering, denoising, merging, and chimera removal was performed with default settings. After data preprocessing, 56,518 reads of 74,938 raw reads (75%) remained in the data set. The resulting amplicon sequence variants (ASVs) were taxonomically classified using the classify-sklearn method and the SILVA SSU database release 128. The data set was further curated by removing ASVs occurring in negative controls (i.e., PCR and DNA extraction blanks, 100 ASVs) and classified as unassigned on the domain level (9 ASVs) or present as singletons (1 ASV). The ASVs in the final data set (752 ASVs represented by 55,187 reads) were collapsed at different taxonomic levels to infer relative sequence abundances. The dominant ASVs of interest were further analyzed by BLASTN comparison against the NCBI nt/nr and ref-seq databases (July 2021), and other selected reference sequences (i.e., “*Candidatus* Arcobacter sulfidicus”), to identify similarity to environmental sequences and cultured representatives.

### Catalyzed reporter deposition fluorescence *in situ* hybridization.

A subsample was fixed in 2% paraformaldehyde for 2 h at room temperature, washed twice with 1× phosphate-buffered saline (PBS) (pH 7.4), and aliquots were stored in 1× PBS at 4°C or in 1× PBS/EtOH at −20°C. Aliquots of the fixed sample were spotted onto wells of Teflon-coated microscopy slides, dried, and briefly washed in 80% ethanol. CARD-FISH was performed as previously described (48). In short, for cell wall permeabilization, samples were incubated in lysozyme solution (10 mg ml^−1^ lysozyme powder, 0.1 mol liter^−1^ Tris-HCl, 0.05 mol liter^−1^ EDTA, pH 8) for 30 min at 37°C. Endogenous peroxidases were inactivated by incubation in 0.01 mol liter^−1^ HCl for 30 min at room temperature. The oligonucleotide probe Arc94 (5′-TGCGCCACTTAGCTGACA-3′) was applied with a formamide concentration of 20% in hybridization buffer (13). The catalyzed reporter deposition step was done with Alexa Fluor 594-labeled tyramides. As we observed that the filaments dissolved during treatment with methanol, we omitted any incubation in methanol (i.e., inactivation of endogenous peroxidases was done with HCl instead of methanol plus H_2_O_2_) and limited all ethanol washing steps. Cells were stained with DAPI (4′,6-diamidino-2-phenylindole), and micrographs were obtained with an epifluorescence microscope (DM4B; Leica).

### Data availability.

Sequences have been deposited at NCBI GenBank under BioProject accession number PRJNA691673.
